# Topological feature generation for link prediction in biological networks

**DOI:** 10.7717/peerj.15313

**Published:** 2023-05-09

**Authors:** Mustafa Temiz, Burcu Bakir-Gungor, Pınar Güner Şahan, Mustafa Coskun

**Affiliations:** 1 Department of Computer Engineering, Abdullah Gul University, Kayseri, Turkey; 2 Department of Artificial Intelligence and Big Data Engineering, Ankara University, Ankara, Turkey

**Keywords:** Graph embedding, Machine learning, Link prediction, Protein-protein interaction, Feature generation

## Abstract

Graph or network embedding is a powerful method for extracting missing or potential information from interactions between nodes in biological networks. Graph embedding methods learn representations of nodes and interactions in a graph with low-dimensional vectors, which facilitates research to predict potential interactions in networks. However, most graph embedding methods suffer from high computational costs in the form of high computational complexity of the embedding methods and learning times of the classifier, as well as the high dimensionality of complex biological networks. To address these challenges, in this study, we use the Chopper algorithm as an alternative approach to graph embedding, which accelerates the iterative processes and thus reduces the running time of the iterative algorithms for three different (nervous system, blood, heart) undirected protein-protein interaction (PPI) networks. Due to the high dimensionality of the matrix obtained after the embedding process, the data are transformed into a smaller representation by applying feature regularization techniques. We evaluated the performance of the proposed method by comparing it with state-of-the-art methods. Extensive experiments demonstrate that the proposed approach reduces the learning time of the classifier and performs better in link prediction. We have also shown that the proposed embedding method is faster than state-of-the-art methods on three different PPI datasets.

## Introduction

Graphs (networks) have been widely used to model the associations and interactions (edges) between biomedical entities (nodes). The analysis of biomedical networks provides great insight into explaining various complex biomedical networks such as, long non-coding RNA (lncRNA)—protein interaction networks (Zhang et al., 2018) and drug-disease associations (DDA) networks (Gottlieb et al., 2011; Long et al., 2022). Network embedding methods, which aim to learn internal continuous hidden representations of nodes, have been proposed for the analysis of networks (Yue et al., 2020). They can be used to learn latent vectors in machine learning and data mining models for various downstream tasks such as node clustering, node classification and link prediction (Han et al., 2022). Network embedding methods can simplify the representation of complex networks, but they are based on traditional methods and suffer from high computational costs, such as high dimension and high computational complexity. One of the problems in the analysis of biological networks is the high dimensionality of the embedding matrix, which indicates the topological properties of the network. Another problem is that the use of high-dimensional data in predicting interactions leads to high computational complexity in the machine learning methods for the embedding and prediction task (Xu, 2021). We first present some background about embedding methods, protein-protein interaction networks and present its applications in biological research. There are various studies on embedding methods, which can be divided into three main categories: random walk-based, neural network-based and matrix factorization-based methods. Random walk-based methods (*e.g*., DeepWalk (Perozzi, Al-Rfou & Skiena, 2014), struct2vec (Ribeiro, Saverese & Figueiredo, 2017), node2vec (Grover & Leskovec, 2016)) have been developed to learn node representations by generating ‘node sequences’ through random walks in networks (Bojanowski, 2017). Random walk-based methods are very effective in analyzing complex biological networks. Since these methods use topological information, they can determine the properties of a complex biological network. Therefore, random walk-based methods can be used in downstream tasks of network analysis (Nasiri et al., 2021). Neural network-based methods (SDNE (Wang, Cui & Zhu, 2016) and LINE (Tang et al., 2015)) use graph embedding operations. This type of analysis can be of great use in various biomedical information processing tasks, such as identifying drug repositioning candidates, predicting missing interactions in protein-protein interaction networks and discovering the function of a lncRNA. A growing number of studies are applying graph embedding (network embedding) techniques to graph simplification and analysis (Nasiri et al., 2021). Matrix factorization-based methods (*e.g*., Isomap (Balasubramanian & Schwartz, 2002), Locally linear embedding (Saul & Roweis, 2000), GF (Ahmed et al., 2013)) aim to keep the topological properties and structure of the manifold and factorize a data matrix into lower dimensional matrices latent in the base data matrix (Nasiri et al., 2021). In biological research, Yue et al. (2020) explored how this idea can be used in three biomedical link prediction problems: protein-protein interaction, drug-drug association, and drug-drug interaction. They compile different datasets from popular biomedical databases and use different graph embedding methods for comparison (Yue et al., 2020). You et al. (2010) developed a robust embedding method to predict new interactions by using the topological information of PPI networks. They compare their proposed method with an existing approach and indicate that their method can achieve better performance in sparse PPI networks. Morever, their method is described as very effective for large sparse PPI networks (You et al., 2010). Cannistraci, Alanis-Lobato & Ravasi (2013) proposed a new solution for link prediction by incorporating network embedding methods. They shed light on the fact that network embedding methods for predicting new PPI can play an important role in better understanding biological associations. They compared their method with other embedding approaches. Their method outperformed other embedding methods in link prediction (Cannistraci, Alanis-Lobato & Ravasi, 2013). Ieremie, Ewing & Niranjan (2022) propose a trainable approach called TransformerGO that predicts PPIs using information from the GO (Gene Ontology) graph. They apply the node2vec method to generate feature vectors for GO terms. Then, they use the Transformer model (Vaswani et al., 2017) to learn the semantic similarity between groups of GO terms. They concluded that the proposed method outperforms the classical similarity measures and other models that use a similar method to encode GO terms (Ieremie, Ewing & Niranjan, 2022). Kuchaiev et al. (2009) proposed an algorithm for embedding PPI networks. They predicted new protein interactions and evaluated the confidence in existing interactions. They achieved 90% sensitivity and 85% specificity, and their method can be applied to large-scale network experiments (Kuchaiev et al., 2009). Chen et al. (2023) propose a model called AdaPPI, a convolutional graph network that uses PPI networks to predict functional protein modules. Comprehensive performance evaluations and case studies show that the proposed method significantly outperforms state-of-the-art methods (Chen et al., 2023). Balogh et al. (2022) present a novel approach that uses a machine learning model to perform link prediction in PPI networks. For graph embedding, they use the node2vec and struc2vec methods developed by Yue et al. (2020). Using their proposed method, they achieved a value of 91.5% AUCROC with their proposed method (Balogh et al., 2022).

In this article, we aim to reduce the high dimensionality and time consumption (in terms of embedding time and classifier learning time) for link prediction in biomedical networks. In our experiments, different protein-protein interaction datasets are used for link prediction. First, the proposed embedding algorithm is applied to obtain topological features of networks. The proposed embedding algorithm significantly reduced the running time compared to state-of-the-art methods. Second, the dimension of the vector generated by the embedding algorithm that defines the topological features of networks, is reduced by applying feature generation methods to reduce the prediction time by the machine learning algorithm. Third, a classification method is applied to predict missing interactions and possible interactions between protein-protein interaction data. For link prediction, extensive experiments were performed on three different tissue samples with protein-protein interactions. Experimental results show that the proposed method outperforms state-of-the-art methods in terms of runtime and classification results for each dataset. We experimentally verified that our models can provide the best performance for the link prediction problem.

The rest of this article is structured as follows. First, we introduce terminology, graph embedding, link prediction, feature generation techniques, and the proposed approach. Subsequently, we give a detailed experimental evaluation of the methods used in this study. Finally, we draw a conclusion and provide directions for further research.

## Materials and Methods

### Datasets

The dataset used in this study contains interaction information for biological networks representing human protein-protein interactions (PPI) in a tissue. In the undirected and unweighted PPI network, nodes represent human proteins that are specifically active in that tissue, and an edge indicates a biological interaction between a pair of proteins. Here we consider the PPI prediction task as a link prediction problem. If there is an interaction between proteins, it is represented as a positive interaction; otherwise, it is represented as a negative interaction. Negative interactions were generated by random pairing of proteins. In this study, we use three different tissue PPI datasets (Nervous System, Blood, Heart) obtained from Stanford Network Analysis Project (SNAP) compiled by Zitnik & Leskovec (2017) (http://snap.stanford.edu/ohmnet/). The nervous system tissue dataset contains 3.533 unique proteins with 54.555 positive interactions, the blood tissue dataset contains 3.316 unique proteins with 53.101 positive interactions and the heart tissue dataset contains 3.201 unique proteins with 48.719 positive interactions. The descriptive statistics of these datasets are shown in Table 1.

**Table 1:** Descriptive statistics of the networks used in the experiments.

Name of tissue	# of nodes	# of interactions
Nervous system	3.533	54.555
Blood	3.316	53.101
Heart	3.201	48.719

## Existing methods

This study focuses on reducing the embedding time with an effective embedding algorithm and reducing the learning time of the classifier by reducing the dimension of the embedding matrices. It also improves the success level in comparison with state-of-the-art methods by using the information from PPI to exploit the downstream tasks such as link prediction. In this section, (i) graph embedding, (ii) link prediction, (iii) feature generation are explained.

### Graph embedding

Let G = (V, E) be an unweighted and undirected graph where V symbolizes the set of vertices (nodes) and E is the set of interactions (edges) in this network. For this network, a network embedding is defined as a matrix H $\epsilon$ ${R^{(nxd)}},$ where *n* = $\mid\!\! V\!\!\mid$. Here, $d$ is a parameter defining the number of dimensions in the embedding feature space. Each row of this matrix (H) represents the embedding of u as ${h_u}$ $\epsilon$ R $^d$ for each node u $\epsilon$ V. The task of graph embedding is to map this graph into a continuous latent space for a given *d* dimension (Coşkun & Koyutürk, 2021).

### Baseline graph embedding methods

For baseline graph embedding methods, we use BioNEV (https://github.com/xiangyue9607/BioNEV) developed by Yue et al. (2020) to learn node embedding for single value decomposition (SVD) (Dai et al., 2015), graph auto-encoders (GAE) (Kipf & Welling, 2016), graph representation (GraRep) (Cao, Lu & Xu, 2015), large-scale information network embedding (LINE) (Tang et al., 2015), graph factorization (GF) (Ahmed et al., 2013). These methods were preferred because high performance metrics were obtained using these approaches in BioNEV. In this section, we provide a brief overview of the different graph embedding methods.

Graph embedding methods are basically divided into three groups. Random walk-based embedding methods: this method starts with a particular initial node and then randomly selects one of its neighbors. This process is repeated for all nodes to obtain node sequences. These sequences are used to uncover hidden information. DeepWalk, struc2vec and node2vec are the algorithms of this method. Matrix factorization (MF)-based embedding methods: this method aims to transform large dimensional matrices used as input data into low dimensional matrices. The topological properties are preserved. SVD, GraRep and HOPE are the algorithms for this method. Neural network-based methods: various neural networks such as Graph Convolutional Network, Autoencoder and Generative Adversarial Network have also been extensively used for graph embedding methods recently. Embedding is performed using different types of graph information as input and different neural architectures. LINE, GAE and SDNE are the algorithms for this method (Yue et al., 2020). GF (graph factorization) learns a low-rank factorization for the adjacency matrix, minimizing the loss of graph regularization. Instead of using the Laplacian matrix, which focuses on factorization, GF directly uses the adjacency matrix to capture first-order proximity (Song et al., 2022). GAE (Graph Autoencoder) encodes both MLP-based and RNN-based methods and utilizes the GCN structure for this encoding. Also, GAE is an unsupervised framework that uses both topological and content information (Pan et al., 2018; Song et al., 2022). LINE is an efficient graph embedding method that transfers real-world problems into a graph structure while preserving them in a scalable way. It is optimized with the Kullback-Leibler metric (KL) by combining first and second order affinities. It uses a sigmoid function for the first order objective and another function for the second order objective. LINE computes the approximations and factorizes them comprehensively (Song et al., 2022). GraRep evaluates the high-order proximity of the network and generates k-step transition probability matrices for factorization. It also uses the node transition probability matrix to capture the similarity of high value nodes (Song et al., 2022). SVD is an MF-based embedding method and is described in detail in the (Lepolesa, Achari & Cheng, 2022).

### Proximity matrix generation

Proximity matrices are used to encapsulate information about the node’s closeness to one another. Various embedding methods use different proximity matrices. A more detailed overview of the proximity matrices used in the network embedding context, can be found in Coşkun & Koyutürk (2021).

In this article, we choose the random walk with restart (RWR)-based proximity matrix as our proximity matrix as it can capture multi-facet relationships among the nodes. In other words, the RWR-based proximity matrix encodes different path associations among the nodes. To compute this RWR-based proximity matrix, we have used the Chopper algorithm (Coskun, Grama & Koyuturk, 2016) (http://compbio.case.edu/chopper/).

The Chopper algorithm has been designed to efficiently compute random walk restarts algorithms. The basic idea behind Chopper is that, in the random walk procedure, remembering the walk that passes through in each node, *i.e*., power method based approaches only rely on the one previous iteration step, while Chopper remembers all previous iterations (walk). To enable this remembering walk, the Chopper utilizes Chebyshev Polynomials, please see Coskun, Grama & Koyuturk (2016), for more details. Overall, Chopper and random walk with restarts algorithms compute the same proximity matrices, however, Chopper is much more efficient than power method-based iterative approaches. In essence, the Chopper algorithm computes the same proximity matrix with random walk restart procedure. The advantage of Chopper is that it computes the RWR-based proximity matrix much more efficiently than that of iterative methods (Coskun, Grama & Koyuturk, 2016) by remembering the paths visited by the random walker. To do so, the Chopper algorithm utilized Chebyshev Polynomials and eventually computes the following RWR-based proximity matrix:

(1) $${\bf{W}} = \alpha {({\bf{I}} - (1 - \alpha ){D^{ - 1}}{\bf{A}})^{ - 1}},$$

where ${\bf{I}}$ denotes the identity matrix, $\alpha$ is the damping factor, which is set to 0.15 (Coskun, Grama & Koyuturk, 2016), ${\bf{D}}$ is degree matrix that contains degrees of each node in its diagonal, and ${\bf{A}}$ is the adjacency matrix. Overall, in this article, we use this RWR-based proximity matrix, ${\bf{W}}$, to generate features from it.

### Link prediction problem

Link prediction deals with the computation of the likelihood that two given nodes will obtain an edge (potential interaction). Link prediction is useful for discovering previously unknown interactions and for identifying missing or spurious interactions. Link prediction is widely used in various biomedical tasks, such as protein-protein interaction prediction, drug-disease association prediction, drug response prediction and drug-drug interaction prediction (Coşkun & Koyutürk, 2021). In this article, the model, trained and developed using information on protein-protein interactions, is used as an input for link prediction.

### Feature generation techniques

The main idea behind the proposed feature generation methods is to determine a small set of entities (nodes) that can be used to represent the position and topological properties of nodes in the network. We use feature generation techniques to reduce the high-dimensional vector and express the information of the graph in smaller dimensions. In this study, we use the Pearson correlation (Pearson, 1896), Lasso regression (L1 Norm), Ridge Regression (L2 Norm) (Golub & Von Matt, 1997) and Kullback Leibler (KL) divergence method (Kullback & Leibler, 1951) as regularization techniques to obtain topological features of nodes from the original embedding data. The high dimensional proximity space is represented by these values, which express the topological features of nodes. In machine learning applications, the Pearson correlation coefficient is a significant method used to measure the similarity of multiple data variables. Pearson correlation coefficient is computed as follows:

(2) $${\rho _{X,Y}} = {{cov(X,Y)} \over {{\sigma _X}{\sigma _Y}}} = {{E((X - {\mu _X})(Y - {\mu _Y}))} \over {{\sigma _X}{\sigma _Y}}}$$

where cov(X,Y) is the covariance between the variable node X and node Y, also ${\sigma _X},{\sigma _Y}$ are standard deviations of variable node X and node Y. E(X) is the expected value of node X.

L1 norm is defined as the sum of the magnitude of the vectors in a space. One of the most efficient methods of measuring the distance between vectors is the sum of the absolute differences of the components of the vectors. The L2 norm is the best-known norm type. It is defined as the shortest distance between two points and all components of the vector are squared. The ${L_p}$ norm can be calculated as follows:

(3) $$||x||{_p} = \root p \of {\sum\limits_{i = 1}^n \mid \!{x_i}{\mid ^p}}$$

${L_1}$ norm, written as $||x||{_1}$ for *p* = 1, is defined as follows:

(4) $$||x||{_1} = \sum\limits_{i = 1}^n \mid \!{x_i}\!\mid$$

${L_2}$ norm is defined as follows for *p* = 2:

(5) $$||x||{_2} = \sqrt {\sum\limits_{i = 1}^n \mid \!{x_i}{\mid ^2}}$$

The Kullback-Leibler (KL) divergence was proposed by Kullback & Leibler (1951). The KL divergence can be used to measure the difference between two probability discrete distributions; and calculated as follows:

(6) $$KL({\rho _X}) = \int {{\rho _Y}(u)ln{{{\rho _Y}(u)} \over {{\rho _X}(u)}}} du$$

where m-dimensional random vectors X and Y have densities ${\rho _X}$ and ${\rho _Y}$ in the network, respectively.

## Proposed method

**Figure 1:**
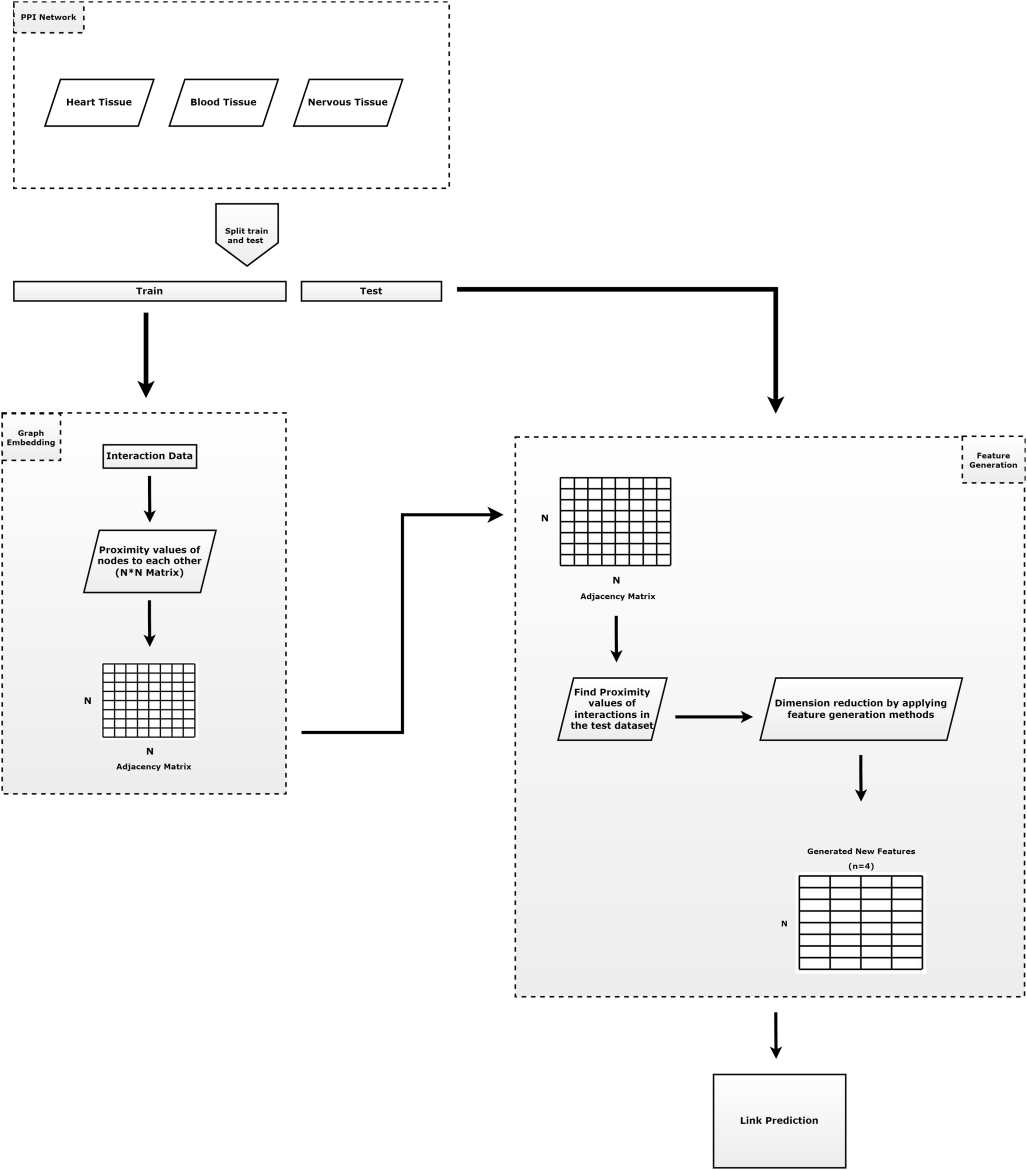
The general approach of the proposed method. It consists of three parts. The first component consists of the input data and the preprocessing of that data. The second component is the step of applying the embedding algorithm. The third component includes the application of feature generation.

Figure 1 summarizes the outline of our proposed method. As shown in Fig. 1, our method is composed of four main tasks: (i) prepare train and test dataset; (ii) learning of the embeddings; (iii) apply feature regularization techniques to feature generation; and (iv) prediction of the links. The contributions of the proposed model can be summarized as follows.

- Protein-protein interactions are split into test set (30%) and training set (70%). Here, all known interactions are considered as positive interactions. If there is an interaction between the node pairs, it is represented as a positive interaction, otherwise as a negative interaction. Since the number of positive node pairs is much lower than the number of negative node pairs, negative interactions are randomly selected to be used for testing phases, with the same number of positive interactions.
- In order to generate structural proximities, the proposed RWR-based embedding algorithm (Chopper) is applied to the training set to obtain a faster value for network proximity values, which reduces the running time. For each pair of nodes we assign the label ‘1’ if the pair has an edge in the training network. However, we assign the label ‘0’ if there are no edges between the nodes.
- Feature regularization methods (Pearson correlation, KL divergence, ${L_1}$ norm and ${L_2}$ norm) are applied to this network proximity matrix to generate intrinsic features with much lower dimension. Thus, a N * N dimensional vector (N: number of nodes) containing the proximity values is reduced to a four-dimensional vector for each node.
- The learned new vectors are utilized as inputs in a logistic regression classifier to predict new interactions. This classifier divides feature scores into train set (70%) and test set (30%).

Previous approaches to link prediction typically use RWR-based network proximity measures to evaluate the interaction between node pairs (Valdeolivas et al., 2019). For the networks, we evaluate the performance of the algorithms using randomized test and training sets. The randomized test sets are repeated three times for each algorithm. For each randomized test set, we select a certain test ratio of interactions (edges) in the network uniformly at random, remove these interactions from the network, and reserve them as positive test sets. Then we compute the node embeddings and perform the training on the remaining network.

### Implementation of the proposed method

The proposed method in this study aims to uncover hidden, unknown, and possible interactions from already known interactions using PPIs. The experiments are performed using Python and Matlab programming languages. This approach can be used for interaction analysis between different data types and processes. The damping factor and restart vector for the Chopper algorithm can be configured by the user. After reading the entire dataset, non-interaction nodes are generated from known interactions. The data is split into a training set and a test set, then the test set is removed from the entire data set. The Chopper algorithm, an embedding algorithm, is applied to the training set to obtain the proximity values between nodes (proteins). Then, using these proximity values, feature generation techniques are applied to the interactions in the test set.

## Results

In this section, we comprehensively evaluate the performance of the proposed method. We first present the experimental settings and then compare the proposed method with state-of-the-art-methods in terms of time consumption (embedding time and learning time of classification algorithm) and link prediction performance. In the experiments, we plotted the time-consuming criteria, accuracy, AUC and F1-measure scores to display the performance of the proposed method.

### Experimental settings

In this section, we perform extensive experiments to verify the proposed method for link prediction. All experiments mentioned in this article were performed on a machine with Intel(R) Core(TM) i7-4710HQ 2.50 GHz, 4 core CPU, 16 GB RAM. The Chopper algorithm is implemented in Matlab programming language, and the feature regularization techniques and preprocessing steps are implemented in Python programming language version 3.8. We randomly split all known PPI pairs into the training and testing dataset. To better train the models, a negative sampling strategy is used. Negative PPI information was randomly sampled from the unknown PPI informations and an equal number of negative and positive informations are used for the model training and testing phases of the model, as proposed in Long et al. (2022). The following parameters are used while running the Chopper algorithm:

- The dimension is set to N × N (N: number of nodes)
- The damping factor ( $\alpha$) is set to 0.85
- The restart vector (rq) is set to 1 at its qth entry and 0 at all other entries

The parameters of the regularization techniques are used as default values. To evaluate the performance of the proposed method in terms of embedding time and prediction time, we also use seconds as the unit of time. Logistic regression method was used as the classifier and experiments were performed with default parameters. To evaluate the performance of the proposed methods, the area under ROC curve (AUROC) values were also used. The experiments were performed with test ratios of 30%. Three different AUROC scores were obtained by repeating the procedures three times.

### Performance evaluation

In this section, we evaluate the performance of the proposed method in terms of time consumption (embedding learning and classification learning) and link prediction performance. In this evaluation, we use embedding methods such as SVD, GAE, GF, GraRep, LINE, which are among the traditional methods widely used in the literature. Graph embedding algorithms are time consuming as they represent high-dimensional data with graphs and convert these structures into vector form. Therefore, we first evaluate the superiority of the proposed method in terms of embedding time. After graph embedding, the proposed approach reduces the size of the input data by creating new features. After dimensionality reduction with feature generation techniques, the learning time of the classifier and the performance of machine learning in link prediction are also important. Therefore, in addition to the graph embedding times, the learning times of the classifier and the performance of machine learning are also evaluated.

#### Comparative evaluation in terms of embedding learning time

**Figure 2:**
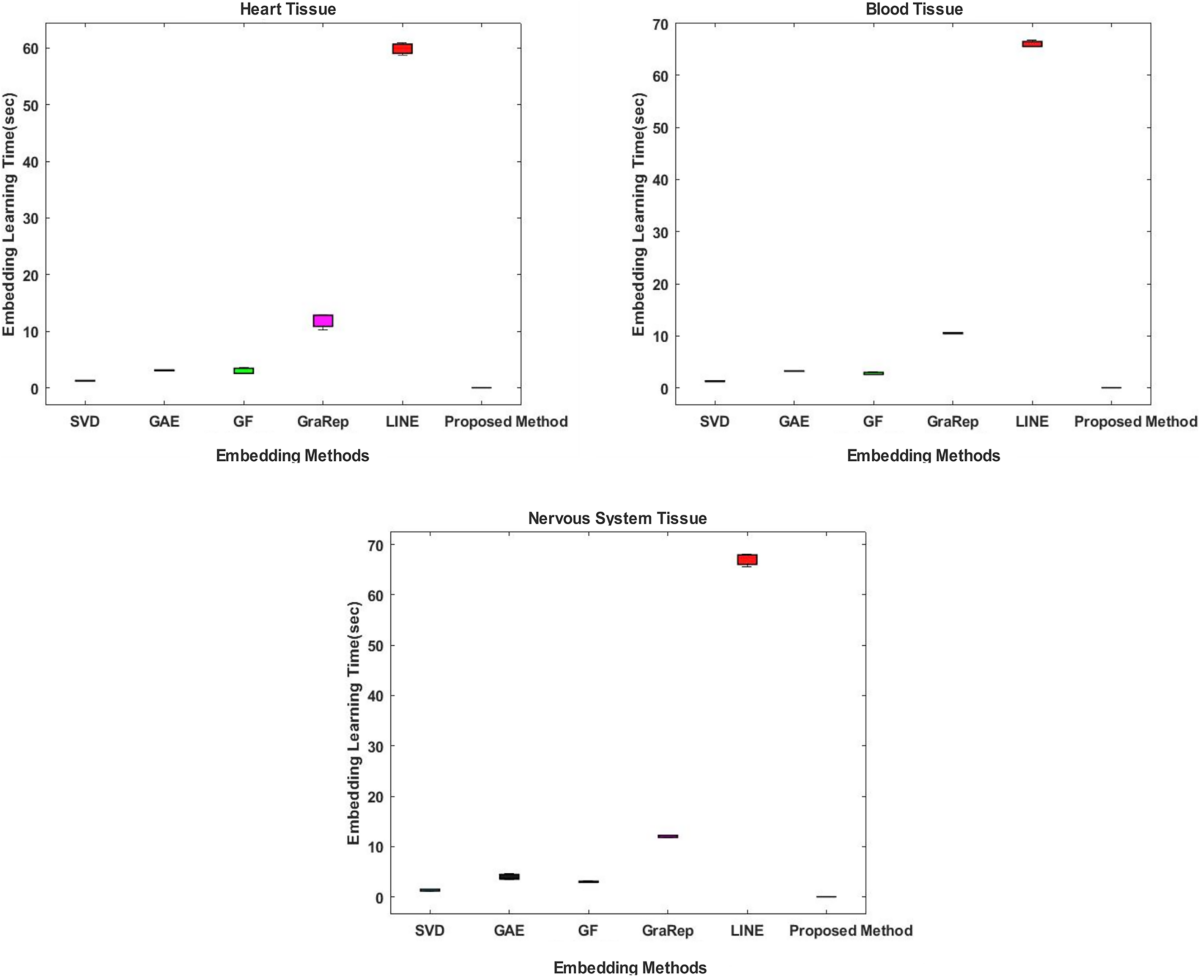
Comparison of embedding learning times in the training process for three different tissue datasets. The x-axis represents the embedding algorithms including the proposed method, and the y-axis represents the learning times in seconds.

In the proposed method, the Chopper algorithm is used as the embedding algorithm. The Chopper algorithm produces a matrix containing the proximity values of nodes to each other. In experiments, the number of rows represents rows for traditional methods, and the number of columns is used with the default value of 100 for all datasets and methods. In the Chopper algorithm, rows and columns also represent the number of nodes. We repeat each experiment three times. The comparison of the embedding learning time of the six methods is summarized in Fig. 2. As can be seen in Fig. 2, the proposed method achieved the best results, which means that it outperforms the other five state-of-the-art methods in all datasets. For heart tissue, the performance of the chopper algorithm is in the range of 0.0039 to 0.0044 s, whereas the SVD method comes closest to the chopper algorithm in the range of 1.22–1.33 s. For blood tissue, the chopper algorithm achieves an embedding learning time of $0.0043 \pm 0.0012$ s, which is nearly 28 times higher than the second best method, SVD. For nervous system tissue, the performance of the chopper algorithm ranges from 0.0046 to 0.0059 s, with the SVD method coming closest to the chopper algorithm in the range of 1.26–1.37 s. Using the appropriate datasets, the longest learning time in terms of embedding learning time is obtained with the LINE algorithm. While the learning times for SVD, GF and GAE are approximately the same with the proposed method, it is highlighted that graph embedding takes a lot of time, especially when using the LINE algorithm and the GraRep algorithm.

#### Comparative evaluation in terms of classifier learning time

While conventional methods classify using 100-dimensional embedding matrices, the proposed method uses four features whose interactions are represented by the embedding matrix. The proposed method shows superior performance on all tissue samples. The most successful results were obtained with the proposed method respectively between 0.054 and 0.155 s for nervous tissue, between 0.046 and 0.187 s for blood, and between 0.045 and 0.165 s for heart.

**Figure 3:**
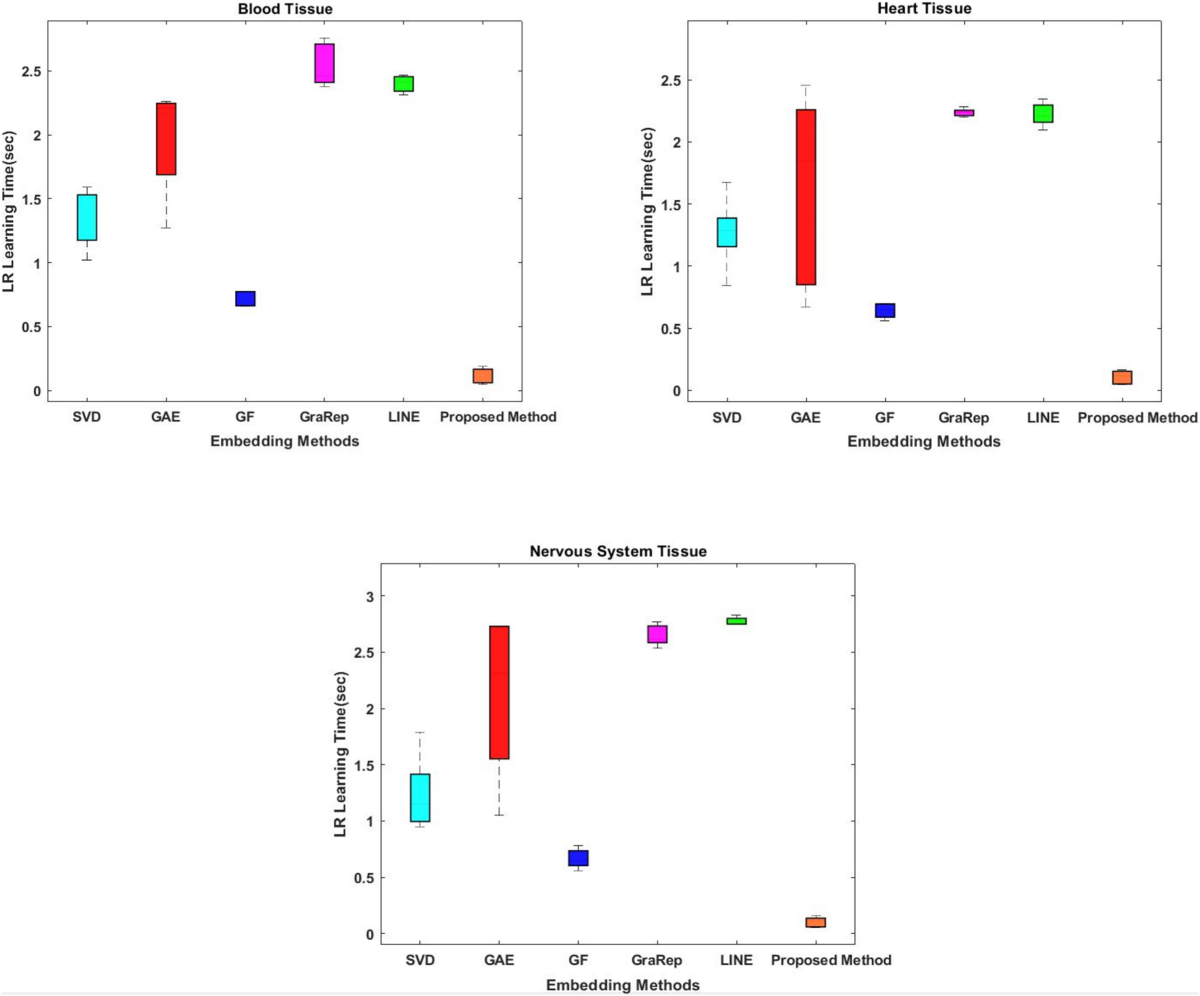
Comparison of learning times of the classifier for three different tissue datasets. The x-axis represents the embedding algorithms and the y-axis represents the learning time of the classifier in seconds for classification using the logistic regression classification method.

Classification prediction time is also important for our model. Therefore, another evaluation criterion is the learning time of the classifier. While conventional methods classify with 100-dimensional embedding matrices as default option, the proposed method uses four-dimensional ones. The interactions of which are represented by the embedding matrix. We attempt to shorten the learning time for classification by obtaining low-dimensional informative data from the high dimensional data. The proposed method shows superior performance on all tissue samples, as shown in Fig. 3. The most successful results were obtained with the proposed method respectively between 0.054 and 0.155 s for nervous tissue, between 0.046 and 0.187 s for blood tissue, and between 0.045 and 0.165 s for heart tissue. The results closest to the proposed method are obtained using the GF algorithm for each tissue sample. Among the other methods compared, the SVD, GAE, LINE and GraRep algorithms are for blood tissue, heart tissue and blood tissue, the SVD, GAE, GraRep and LINE algorithms are for heart tissue and blood tissue, respectively.

#### Link prediction performance

**Figure 4:**
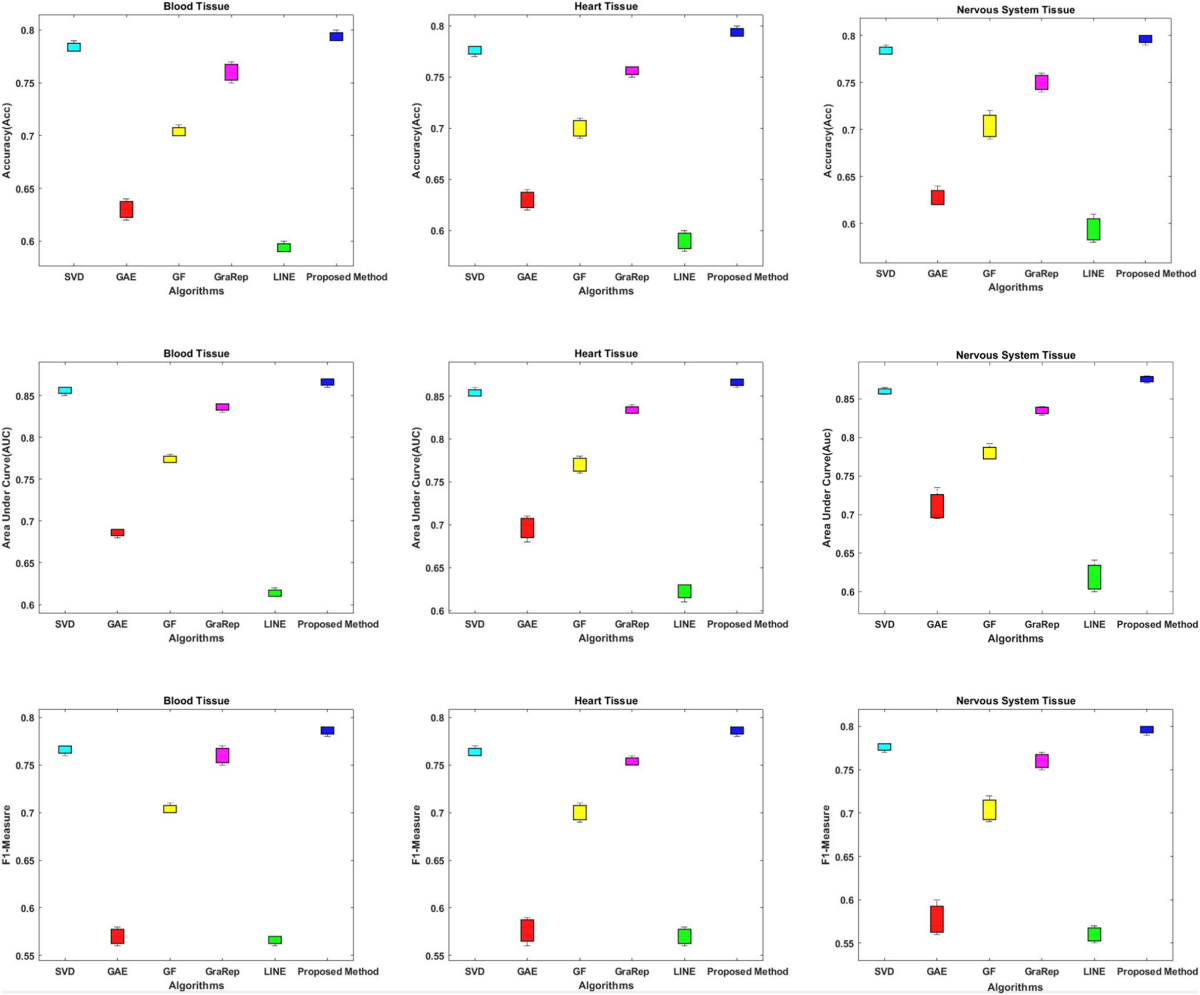
Link prediction performance of graph embedding computed by using different embedding methods. In each figure, the x-axis represents the algorithms (methods) that use embedding on PPIs, the y-axis represents the accuracy, AUC and F1-measure for link prediction.

In this section, we compare the proposed method in terms of AUC, Accuracy and F1-measure scores with different embedding algorithms in biomedical networks. Figure 4 compares the link prediction performance of the proposed method with other embedding algorithms. In Fig. 4, each graph embedding algorithm is represented by a different color. Accuracy, AUC, and F1-metrics are obtained for each method for each tissue network. Each column shows a different algorithm, the first row shows the accuracy values, the second row shows the AUC values, and the third row shows the F1-measure values for different datasets. The AUC value is used to compare the performance of the proposed method with other embedding algorithms.

Based on these results, we can make the following observations.

- The proposed method achieves an AUC of about 86–88% for the blood tissue dataset and an AUC of about 86–89% for the heart tissue dataset. For the nervous system tissue dataset, AUC values between 86–88% are obtained. The proposed method performs better than the other tested embedding algorithms.
- The closest link prediction performance values to the proposed method are obtained using the SVD algorithm, for all tested tissue networks. With the SVD algorithm, an AUC value of about 85–87% is obtained. Following the SVD algorithm, the next best results are obtained with the GraRep, GF, GAE, and LINE methods, respectively.
- In addition, the AUC confidence intervals at the 95% level are (0.857, 0.863) for blood tissue, (0.877, 0.883) for heart tissue, and (0.867, 0.873) for nervous system tissue.

## Discussions

In this article, we propose a method based on embedding algorithms and feature generation methods to solve the link prediction problem in biomedical networks. We have comparatively analyzed our method with other graph embedding and feature generation methods for the link prediction problem. The experiments were performed on three different tissue datasets that contained information about protein-protein interactions in different tissues. We have extensively tested embedding methods such as Chopper, SVD, GAE, GF, GraRep and LINE in terms of time consumption; and feature regularization techniques in terms of dimension reduction and learning time of the classification method. Our experiments show that the proposed method outperforms the state-of-the-art methods in terms of embedding time and classification learning time. The graph embedding is achieved in shorter time with the proposed approach, as shown in Fig. 2. Also, the learning times of different classifiers are compared in Fig. 3. Both time analyzes demonstrated that the proposed approach outperforms the compared methods. Moreover, different performance metrics achieved by the proposed approach and by other approaches are comparatively evaluated in Fig. 4. As this figure implies, our approach provides better link prediction performance than state-of-the-art methods. With the proposed approach, more accurate results can be achieved in shorter time. With this approach, the transition from high-dimensional data to low-dimensional data is achieved. As a future work we consider the following tasks: (i) The proposed method will be applied to heterogeneous biological samples (protein-drug, disease-drug, protein-disease); (ii) the Chopper algorithm will be updated, new technologies will be integrated; (iii) We plan to create a web tool to retrieve the results simply by uploading the input data, which can facilitate the usage of the tool by the molecular biologists and geneticists.

## Conclusion

In this article, we propose a link prediction method based on proximity measures and feature generation methods that offers lower computational costs. We evaluate this approach on several biological data. Three different biological tissue data containing PPI information are extensively compared with the performance of the proposed method using five different embedding methods. These experiments highlight the better performance of the proposed method. Therefore, the proposed approach can be used and evaluated in biomedical studies with complex interaction data. The proposed method will be used in the diagnosis and treatment of common diseases by integrating its parameters and interacting structures with current technologies. In this way, this approach is expected to contribute to low computational cost and high accuracy of diagnosis and treatment time.

## Abbreviations

AUC: Area under the ROC Curve
ROC: Receiver Operating Characteristic
LR: Logistic Regression
PPI: Protein-Protein Interaction
RWR: Random Walk with Restart
SVD: Singular Value Decomposition
GraRep: Graph Representation
GAE: Graph Auto-Encoders
GF: Graph Factorization
LINE: Large-scale Information Network Embedding
BioNEV: Biomedical Network Embedding Evaluation
KL: Kullback-Leibler
L1 Norm: Lasso Regression
L2 Norm: Ridge Regression

## Additional Information and Declarations

### Data Availability

The following information was supplied regarding data availability: The code is available at GitHub and Zenodo: https://github.com/MstafaTmz/LinkPreDict. Mustafa Temiz. (2023). MstafaTmz/LinkPreDict: 1.0.0 (v1.0.0). Zenodo. https://doi.org/10.5281/zenodo.7811879.
